# Antimicrobial Effect of Postbiotics on Multidrug-Resistant *Escherichia coli*

**DOI:** 10.3390/foods15020384

**Published:** 2026-01-21

**Authors:** Çiğdem Sezer, Nebahat Bilge, Gönül Damla Büyük, Merve Ayyıldız Akın

**Affiliations:** 1 Department of Food Safety and Public Health, Faculty of Veterinary Medicine, Kafkas University, 36100 Kars, Türkiye; 2 Department of Biostatistics, Faculty of Veterinary Medicine, Kafkas University, 36100 Kars, Türkiye

**Keywords:** postbiotics, multidrug-resistant *Escherichia coli*, *Lactiplantibacillus plantarum*, *Lactococcus lactis*

## Abstract

Pathogens that have developed resistance to antibiotics pose a threat to public health. The primary goal in preventing foodborne infections is to inhibit the growth of and, subsequently, eliminate antibiotic-resistant pathogens at every stage from production to consumption. Escherichia coli, which has acquired resistance to most known antibiotics, is frequently found in chicken meat. In many countries, due to unregulated antibiotic use in poultry farming, poor hygiene in slaughterhouses, or cross-contamination, extended-spectrum beta-lactamase (ESBL)-producing *E. coli* has been identified as the causative agent in poultry-associated food poisoning. The need for more effective antimicrobial agents against this pathogen, which is resistant to existing antibiotics, has led to increased attention being paid to postbiotics produced by lactic acid bacteria, particularly bacteriocins. This study aimed to determine the antimicrobial effects of postbiotics obtained from kefir-derived *Lactiplantibacillus plantarum* and *Lactococcus lactis* against ESBL-positive *E. coli*. To achieve this, *E. coli* strains were isolated from raw chicken meat samples collected from the market using culture-based methods, and their antimicrobial resistance profiles were determined using the disk diffusion method. The ESBL positivity of the isolates was assessed using the double-disk synergy test. The antimicrobial activities of the postbiotics against the identified ESBL-positive *E. coli* strains were tested using the macro-dilution method to determine minimum inhibitory concentration (MIC) and minimum bactericidal concentration (MBC) values. ESBL-positive *E. coli* was detected in 48% of raw chicken meat samples. The antimicrobial effects of postbiotics were examined by disk diffusion, and postbiotics produced by 18 *Lb. plantarum* strains and 20 *Lc. lactis* strains showed strong antimicrobial activity. Significant differences in the antimicrobial effects of postbiotics were observed between the two species. *Lb. plantarum* postbiotics exhibited both bacteriostatic (concentration 60%) and bactericidal (concentration 80%) effects on ESBL-positive *E. coli* strains, whereas *Lc. lactis* postbiotics showed only bacteriostatic effects (80% concentration). Postbiotics derived from probiotic bacteria offer promising effects against multidrug-resistant *E. coli* due to their heat resistance, activity across different pH values, strong antimicrobial effects, affordability, and ease of production.

## 1. Introduction

Antimicrobial resistance is a global problem for public and animal health in both developed and developing countries worldwide. It is known that antibiotic-resistant microorganisms can spread through animal products and humans [1]. If effective strategies to slow the increase in antimicrobial resistance are not found, it is estimated that millions of people could be at risk of death in the coming decades due to the rise in antimicrobial-resistant infections [2]. Antimicrobials are used worldwide, particularly in poultry, to treat bacterial infections, prevent infections, and promote growth [1,3]. The European Union prohibited the prophylactic use of antibiotics in animal feed in 2006 [4]. In the United States, one of the largest poultry producers, the FDA, has taken several key measures to ensure the appropriate and rational use of medically important antimicrobial drugs in poultry. In its 2015 veterinary feed directive, the FDA prohibited the prophylactic use of antibiotics and stipulated that antimicrobials can only be administered with a veterinary prescription [5]. Despite these regulatory measures, antimicrobial-resistant bacteria and the infections they can cause, which represent a global threat, endanger both public health and food safety. Although the OECD-FAO Agricultural Outlook 2022–2031 report states that poultry meat will lead the growth of the meat sector [6], many studies have shown that antibiotic-resistant pathogens in chicken meat are a major public health problem. The presence of extended-spectrum beta-lactamase (ESBL)-producing *E. coli* in chicken meat has been reported in numerous studies [7,8,9,10,11,12,13]. The presence of such high levels of antimicrobial-resistant microorganisms in chicken meat, which is a popular food, poses a considerable risk to public health. Tsitsos et al. (2025) investigated the presence of antibiotic-resistant *E. coli* in chicken meat and examined the relationships of the isolates obtained according to regions and seasons [13]. Through protein profile analysis, they revealed the relatedness of isolates obtained in different seasons and regions. Based on these results, they emphasized that poultry meat is a very important reservoir for resistant *E. coli* and that all necessary hygiene measures should be taken to protect public health [13]. Schwaiger et al. (2012) reported that antimicrobial-resistant *E. coli* were predominantly found in chicken meat samples collected from poultry slaughterhouses and retail outlets, and that it was difficult to determine the precise stage in the production chain at which contamination occurred [14]. Kim et al. (2020) investigated the presence of antimicrobial-resistant *E. coli* in chicken meat sold in Korea and obtained noteworthy results [15]. In their study, chicken meat from standard poultry farms, as well as meat labeled as organic or antibiotic-free, was included in the research groups. According to the study data, antimicrobial-resistant *E. coli* were found in high amounts in all three groups of chicken meat. Based on these findings, it was concluded that the contamination likely originated from the slaughterhouse [15]. A similar study was conducted in Canada by Chalmers et al. (2021) [16]. Ceftiofur use in poultry farming was prohibited in Canada in 2014; yet several years after this, these researchers isolated extended-spectrum cephalosporin-resistant *E. coli* from the feces of chickens raised with and without therapeutic antimicrobials for two years and noted that both rearing methods yielded similar results [16]. Projahn et al. (2019) investigated cross-contamination of antibiotic-resistant *E. coli* before and after slaughter in two different broiler flocks (ESBL-negative) that were monitored and analyzed at a slaughterhouse [17]. Through phylogenetic analysis, they determined that the slaughterhouse (particularly the feather-plucking and scalding water stages) was a significant source of contamination during poultry processing [17]. Zhao et al. (2024) conducted a study analyzing the genes of antimicrobial-resistant *E. coli* in retail chicken meat [18]. Their comparative analysis of chicken meat-derived and human-derived *E. coli* revealed that plasmids carrying *mcr-1* or *blaNDM* from *E. coli* obtained from chicken meat showed high similarity to plasmids obtained from human-derived *Enterobacteriaceae*. This result clearly demonstrates the risk of *mcr-1* or *blaNDM* transmission from retail meat to humans. The simultaneous presence of *mcr-1* and *blaNDM* in *E. coli* highlights a serious threat to public health and emphasizes the importance of antimicrobial resistance surveillance in retail meats [18]. The WHO states that antimicrobial resistance is one of the top 10 public health threats facing humanity and emphasizes the vital importance of conducting research to discover and develop new antimicrobials [19]. Two possible solutions have been envisioned for the problem of antimicrobial resistance in pathogens for which known antibiotics are ineffective: either more potent chemicals/antibiotics will be discovered, or very detailed research will be conducted on the usability of natural antimicrobial compounds. To this end, research has long focused on bacteriocins and postbiotics produced by lactic acid bacteria. Different data exist regarding the stability (temperature, pH, enzyme, etc.), antimicrobial activity, mode of action (Gram-negative or Gram-positive cell structure), and application method of each bacteriocin [20,21,22,23]. Although the effects of postbiotics depend on several factors, such as the target microorganism and the method of application, the most critical factor to be considered is the accurate selection of the bacteriocin-producing strain. The primary objective of this study is to isolate lactic acid bacteria with antimicrobial activity (*Lb*. *plantarum* and *Lc. lactis*) from kefir, a probiotic food containing a wide variety of lactic acid bacteria, and to evaluate the antimicrobial effects of the postbiotics produced by these bacteria against ESBL-producing *E. coli*. In addition, when selecting alternative antimicrobial agents for the food industry, not only strong antimicrobial activity but also economic feasibility and practical applicability are of great importance. In many similar studies aiming to develop alternative antimicrobials, the focus has been on bacteriocins and their purified forms. In contrast, this study investigates the direct applicability of postbiotics without the need for bacteriocin purification, focusing on their stability under different heat treatments, resistance over time, and related practical properties.

## 2. Materials and Methods

### 2.1. Materials

In the study, samples of fresh-cut (non-frozen) chicken thighs and/or wings from different brands and batch numbers were collected from ten retail outlets. A total of 100 samples were transported to the laboratory under cold-chain conditions as quickly as possible, and the samples were kept at 4 °C throughout the study period. Kefir samples were produced under laboratory conditions using UHT milk and kefir grains obtained from five different sources (3%, *w*/*v*). Fermentation was carried out at 30 °C for 18, 24, and 48 h, and the resulting kefir samples were used for further analyses.

### 2.2. Methods

#### 2.2.1. Isolation and Identification of *E. coli* from Chicken Samples

For the isolation of *E. coli*, at least 50 g of each chicken sample was homogenized with nine volumes of Tryptic Soy Broth (TSB) to prepare decimal dilutions. Aliquots were spread onto two Violet Red Bile Lactose Agar (VRBA) plates. One plate was incubated at 44.5 °C and the other at 35–37 °C for 18–24 h. Following incubation, typical colonies were purified and stored as stock cultures. Identification was performed using conventional biochemical tests, including Gram staining, oxidase, catalase, and IMViC tests [24]. *E. coli* ATCC 25922 was used as a positive control.

#### 2.2.2. Isolation and Identification of *Lb. plantarum* and *Lc. lactis* from Kefir

In our previous study [25], bacteriocins produced by *Lb. plantarum* and *Lc. lactis* among lactic acid bacteria were found to exhibit broad-spectrum antimicrobial activity. Therefore, the isolation of these two lactic acid bacteria was specifically targeted in this study.

In this study, different kefir samples were obtained using kefir grains from five different sources. The kefir grains were fermented in sterile milk at 30 °C, and samples were taken from the kefirs at the 18th, 24th, and 48th hours of fermentation (to enhance the probability of isolating the target lactic acid bacteria). These samples were then plated onto MRS Agar and M17 Agar media and incubated at 30 °C for 48–72 h. At the end of incubation, typical colonies were selected from each medium, purified, and stored as stock cultures. The obtained isolates were grouped using basic identification tests such as Gram staining, microscopic morphology, catalase reaction, oxidase test, and glucose gas production [26]. Molecular identification was carried out by PCR following DNA extraction using a commercial kit (QIAGEN, QIAamp-51304, Venlo, The Netherlands). The PCR mix and conditions described by Torriani et al. (2001) and Pu et al. (2002) were used with minor modifications [27,28]. For *Lb. plantarum* (amplicon 318 bp.), planF (5′-CCG TTT ATG CGG AAC ACC TA-3′) and pREV (5′-TCG GGA TTA CCA AAC ATC AC-3′) primers were used, while for *Lc. lactis* (amplicon 161 bp.), LacreR (19-GGGATCATCTTTGAGTGAT) and LacF (19-GTACTTGTACCGACTGGAT) primers were used [27,28,29]. In genetic identification tests, *Lb. plantarum* NRRL-B 4496 and *Lc. lactis* CECT 4432, obtained from the ARS culture collection, were used as positive controls. The primers used for the identification of *Lb. plantarum* and *Lc. lactis* are presented in Table 1.

#### 2.2.3. Determination of the Antimicrobial Effects of *Lb. plantarum* and *Lc. lactis* on *E. coli*

The disk diffusion method was used to evaluate the antimicrobial activity of *Lb. plantarum* and *Lc. lactis* against *E. coli*. For this purpose, *Lb. plantarum* and *Lc. lactis* were cultured in MRS broth at 30 °C for 18 h. Lactic acid bacteria cultures were centrifuged at 10.000× *g* for 10 min at 4 °C. The supernatants were collected and sterilized by microfiltration. *E. coli* suspensions in TSB adjusted to McFarland 0.5 were inoculated onto Mueller–Hinton Agar. After inoculating 100 μL of *E. coli* strain cultures, 100 μL samples of sterile supernatants of LAB were inoculated onto blank paper disks placed at specific intervals on the Mueller–Hinton Agar surface. Plates were incubated at 35–37 °C for 24–48 h, and after incubation, any zones around the disk were examined, and their diameters were measured [25]. The presence of any inhibition zone was considered indicative of antimicrobial activity. According to the CLSI-2024 and EUCAST-2025 guidelines [30,31], the maximum measurable diameter indicating resistance to various antibiotics was defined as ≥2.7 cm. Therefore, when evaluating the effects of postbiotics, an inhibition zone diameter of ≥2.7 cm was considered to represent a strong antimicrobial effect. The existing antimicrobial effect may be due to low pH value, the possible presence of hydrogen peroxide, or bacteriocins with a protein structure. To differentiate these effects, fresh cultures were prepared separately for *Lb. plantarum* and *Lc. lactis* isolates showing antimicrobial activity, and their supernatants were obtained by centrifugation. The supernatants, which were sterilized by microfiltration, were subjected to pre-treatment (pH neutralization, application of 1 mg/mL catalase to neutralize possible hydrogen peroxide, and application of proteolytic enzymes, including of 1 mg/mL protease), and the antimicrobial effect was retested after treatment using the method described above. Loss of the antimicrobial activity following protease treatment revealed that the antimicrobial effect originated from a protein-structured compound. Following this stage, considering the applicability criteria (practicality and cost) in the poultry sector, the purification processes of the detected bacteriocins were not carried out, and the study continued with sterile supernatants. Furthermore, to determine some of the properties of the supernatants, antimicrobial activity stability tests were applied to the supernatants after different heat treatments (65 °C for 30 min/115 °C for 15 min). After this stage, these neutralized and sterilized supernatants were subsequently referred to as postbiotics [25].

#### 2.2.4. Antimicrobial Resistance Determination Tests

The Kirby–Bauer disk diffusion method was used to determine the antimicrobial resistance of *E. coli* strains isolated from chickens. Antimicrobial susceptibility tests were performed on 16 antibiotic agents representing different antibiotic groups: Meropenem (10 μg), Ciprofloxacin (5 μg), Aztreonam (30 μg), Gentamicin (10 μg), Netilmicin (10 μg), Amikacin (30 μg), Ampicillin (10 μg), Ticarcillin/Clavulanate (75–10 μg), Chloramphenicol (30 μg), Trimethoprim/sulfamethoxazole (125–23.75 μg), Cefoxitin (30 μg), Cefotaxime (5 μg), Cephalothin (30 μg), Cefpodoxime (10 μg), Ceftazidime (10 μg), and Ceftriaxone (30 μg). Fresh cultures of the test strains were prepared in saline solution with concentration adjusted to McFarland 0.5 (approximately 1–2 × 10^8^ cfu/mL) and plated on Mueller–Hinton agar using a sterile swab. After plating, antibiotic disks were also placed on the plates, and the plates were incubated at 35 °C for 18 ± 2 h [32]. At the end of incubation, zone diameters were measured and evaluated according to CLSI-2024 and EUCAST-2025 data [30,31]. *Klebsiella pneumoniae* ATCC 700603, *E. coli* ATCC 35218, and *E. coli* ATCC 25922 were used as control strains in the tests. In *E. coli* strains showing resistance as a result of antibiotic resistance tests, the presence of broad-spectrum beta-lactamase was investigated.

#### 2.2.5. Detection of Extended-Spectrum Beta-Lactamase-Producing *E. coli*

ESBLs are enzymes that hydrolyze most penicillins and cephalosporins, including oxime-beta-lactams, but are unaffected by cefamycins and carbapenems, and are inhibited by clavulanic acid, sulbactam, and tazobactam. The double-disk synergy method was used to detect ESBLs in *E. coli* strains. Cephalosporins ceftriaxone, cefotaxime, ceftazidime, and cefpodoxime were used with amoxicillin–clavulanic acid. The Kirby–Bauer disk diffusion method was used for the test. Fresh cultures of *E. coli* isolates were prepared. Concentrations were adjusted to McFarland 0.5 in physiological saline and inoculations were performed on Mueller–Hinton agar using sterile swabs. After inoculation, ceftriaxone, cefotaxime, ceftazidime, and cefpodoxime, along with amoxicillin–clavulanic acid disks, were placed in plates, and the plates were incubated at 35–37 °C for 18 ± 2 h [32]. At the end of incubation, zone diameters were measured, and if the zone of any of the cephalosporin disc increased toward the clavulanic acid disc, the result was considered ESBL-positive [33].

#### 2.2.6. Determination of the Antimicrobial Effect of Postbiotics on ESBL-Positive *E. coli*

In this phase of the study, the macro-dilution method was used to investigate the effects of postbiotics, which were found to exert antimicrobial activity against the reference *E. coli* strains ATCC 35218 and ATCC 25922, on *E. coli* isolates obtained from chicken meat samples which were determined to have developed multidrug resistance [34]. For this purpose, multidrug-resistant *E. coli* isolates adjusted to McFarland 0.5 were inoculated (in quantities of 100 µL) into solutions of *Lb. plantarum* and *Lc. lactis* postbiotics prepared in different concentrations (1.25–100%) in Mueller–Hinton broth, and the tubes were incubated at 35 °C. Evaluations were performed at 0, 6, 12, 24, 48, and 72 h of incubation. The lowest postbiotic concentration showing no growth in the tubes was determined as the minimum inhibitory concentration (MIC) value. The tubes showing no growth were subsequently plated on Mueller–Hinton agar, and the plates were incubated at 35 °C for 12–24 h. The lowest concentration showing no bacterial growth after incubation was determined as the minimum bactericidal concentration (MBC) value. For this assay, Mueller–Hinton broth without antimicrobial agent (postbiotic) and without bacteria (*E. coli*) inoculation was used as a negative control. Broth containing bacterial culture and broth containing antimicrobial agents were used as additional control groups.

#### 2.2.7. Statistical Analysis

The G*Power 3.1.7.9 program was used to calculate the required sample size for this study. The minimum sample size was determined as 45 multidrug-resistant *E. coli* isolates, based on a test power of 0.95, an alpha error probability of 0.05, and an effect size of 0.50. All antimicrobial efficacy experiments were performed independently in triplicate. The data obtained in this study were analyzed using IBM SPSS statistics version 25.0. Descriptive statistics analyses were conducted for the evaluation. The normality of data distribution was assessed using the Kolmogorov–Smirnov test, and the result indicated that the data did not follow a normal distribution (*p* < 0.05). Therefore, continuous variables were summarized using median (Q1–Q3) values. Since the variables were categorical and the observations were independent, the Chi-square test—which is appropriate for testing differences in frequencies between categorical variables—was used to evaluate associations between groups. Categorical variables were expressed as frequencies and percentages. In addition to *p*-values, effect sizes were calculated to assess the strength of the associations. Cramer’s V was used as the measure of effect size, and effect sizes were interpreted as small, medium, or large. Descriptive statistics for continuous variables included the median, standard error, Q1–Q3, minimum, and maximum values, while distributions of categorical variables were presented as frequencies and percentages (%). A significance level of *p* < 0.05 was accepted for all analyses.

## 3. Results

A total of 125 *E. coli* isolates were identified in 83 (83%) of 100 raw chicken meat samples. All of these isolates were tested against 16 antibiotic agents. All isolates were susceptible to meropenem but resistant to aztreonam, cephalothin, cefoxitin, and ampicillin. Ampicillin resistance was inhibited by clavulanic acid in 70% of the isolates. Resistance to the fluoroquinolones, aminoglycosides, and other cephalosporins varied among the isolates. The antimicrobial susceptibility of the *E. coli* isolates is shown in Table 2.

**Figure 1:**
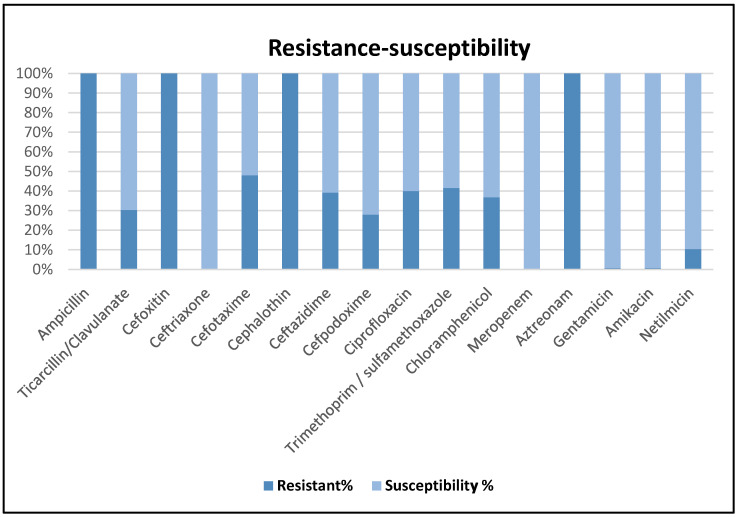
The antimicrobial susceptibility and resistance profile of *E. coli* isolates.

The antimicrobial susceptibility and resistance profile of *E. coli* isolates is presented in Figure 1.

When all isolates were tested for ESBL production using the double-disk synergy method, 57 (45.6%) of the 125 isolates were determined to be ESBL-positive. The distribution rate of ESBL-positive isolates in chicken samples was found to be 48% (48 chicken samples). As a result of the cultural and molecular identification of 382 isolates obtained from kefir samples, 112 were identified as *Lb. plantarum* and 69 as *Lc. lactis*. The antimicrobial effects of these isolates and their postbiotics against *E. coli* used as reference strains were tested by the disk-diffusion method and it was determined that postbiotics produced by 18 *Lb. plantarum* strains and 20 *Lc. lactis* strains exhibited strong antimicrobial activity against the reference *E. coli* strains. A statistically highly significant difference was determined between the antimicrobial activity levels of the postbiotics produced by lactic acid bacteria (X^2^(2) = 178.472, *p* < 0.0001; Cramer’s V = 0.166). The effects of postbiotics on ESBL-positive *E. coli* strains were evaluated and the MIC and MBC values were determined. It was found that postbiotics of *Lb. plantarum* exerted both bacteriostatic and bactericidal effects on ESBL-positive *E. coli* strains, with the MIC value observed at 60% concentration and the MBC value at an 80% concentration. For *Lc. lactis* postbiotics, the MIC value was observed at an 80% concentration; however, this concentration was insufficient to achieve an MBC value. A significant finding was that the antimicrobial effect increased proportionally with increasing postbiotic concentration (X^2^(9) = 4417.18, *p* < 0.0001; Cramer’s V = 0.824).

The concentration- and time-dependent antimicrobial effects of postbiotics produced by lactic acid bacteria are presented in Table 3 and Table 4.

It was determined that *Lc. lactis* postbiotics exerted a bacteriostatic effect; however, considerably higher concentrations would be required to discuss a potential bactericidal effect. When the effect of incubation time on antimicrobial activity was evaluated, it was found that the antimicrobial effect began at the sixth hour of incubation, and prolongation of the incubation period did not result in either an increase or decrease in antimicrobial activity (X^2^(5) = 365.056, *p* < 0.0001; Cramer’s V = 0.237).

To describe the distribution of continuous variables in this study, the median, interquartile range (Q1–Q3), and minimum–maximum values were calculated. Descriptive statistics for these variables are presented in Table 5.

## 4. Discussion

The fact that foodborne illnesses often go unrecognized by healthcare professionals, and that food poisoning is not considered in cases of severe diarrhea, are common issues that hinder a comprehensive understanding of the relationship between contaminated food and public health [35]. According to WHO data, approximately 1 in 10 people worldwide suffer from food poisoning due to the consumption of contaminated food, and 420.000 people die each year as a result of foodborne diseases [36]. Salmonella, campylobacter, and enterohemorrhagic *E. coli*, which cause life-threatening diseases, are among the most common foodborne pathogens. In the modern era, there is a serious problem of resistance to antibiotics used against these pathogens. The unnecessary and excessive use of antibiotics in both human and veterinary medicine has led to the emergence of multidrug-resistant pathogens [36]. The WHO’s 2024 bacterial priority pathogens list includes ESBL-positive *E. coli* among pathogens that develop multidrug resistance [37]. In the present study, ESBL-positive *E. coli* was detected in 48% of the chicken samples. Considering that chicken meat is consumed by individuals of nearly all age groups, a prevalence rate of 48% is notably high. It should be noted that results vary between studies depending on differences in poultry rearing, slaughtering, and processing practices across countries and regions, as well as the sensitivity of the analytical methods employed. Indeed, previous studies have reported ESBL-positive *E. coli* prevalence rates ranging between 10% and 80% [38,39,40]. Randall et al. (2021), in studies conducted at different time intervals, reported that the prevalence of ESBL-producing *E. coli* in chicken meat was 65.4% in 2013, whereas following the implementation of rational antimicrobial use and regulatory measures, this rate decreased to 7.4% in 2018 [39]. The sources of ESBL-positive *E. coli* contamination in chicken meat may vary substantially [39]. Contamination can occur at multiple stages, ranging from evisceration and feather-plucking processes to food handlers and retail sales environments [41,42]. The aim of this study was to propose a natural and effective alternative antimicrobial agent that could be applied in poultry slaughterhouses against antibiotic-resistant *E. coli* isolates. The antimicrobial activity of postbiotics produced by probiotic strains isolated from different kefir samples is of significant interest from a public health perspective. In recent years, the potential applications of postbiotics produced by lactic acid bacteria in the food, pharmaceutical, and healthcare industries have been extensively investigated [43,44,45,46,47]. In this study, bacteriocins were identified as particularly effective postbiotic components due to their colorless and odorless nature, ease and low cost of production, and ability to retain antimicrobial activity following high-temperature treatment. When their antimicrobial effects were tested on *E. coli*, 18 of the 112 *Lb. plantarum* strains and 20 of the 69 *Lc. lactis* strains were found to be antimicrobial. Although previous studies have shown that postbiotics derived from lactic acid bacteria exhibit strong antimicrobial effects primarily against Gram-positive microorganisms, their efficacy against Gram-negative bacteria is generally limited due to the presence of an outer membrane. The antimicrobial action of bacteriocins occurs through disruption of cell wall biosynthesis or by forming pores in the cytoplasmic membrane, leading to impaired membrane permeability and cell death [25,48,49,50]. In the present study, a relatively small proportion of isolates (16% for *Lb. plantarum* and 28.9% for *Lc. lactis*) among a large number of kefir isolates were found to have antimicrobial effects against the *E. coli* reference strains and ESBL-positive *E. coli* isolates. The antimicrobial effect may be influenced by external factors such as the pH, temperature, salt concentration of the food/environment, as well as the cell wall structure characteristics of the target microorganism. The robust cell wall structure of Gram-negative bacteria poses a significant barrier to bacteriocin activity. Apart from the Gram-negative cell wall structure, differences in the structures of the bacteriocins themselves also indicate that their antimicrobial effect occurs through different mechanisms. Bacteriocins exert their effect by binding to Lipid II and/or the mannose phosphotransferase system in the cell wall, thus causing the formation of pores in the cell membrane during the peptidoglycan biosynthesis mechanism [51,52,53,54,55,56]. In terms of antimicrobial efficacy, the length of the incubation period (short or long) did not cause any change in the increase or decrease in antimicrobial efficacy. The antimicrobial efficacy observed within the first 6 h persisted throughout the 72-h incubated period. This finding suggests that postbiotics may exhibit antimicrobial performance comparable to that of conventional chemical antibiotics. We found that the antimicrobial efficacy on ESBL-positive *E. coli* isolates was both bacteriostatic and bactericidal in *Lb. plantarum* postbiotics, while the efficacy was only bacteriostatic in *Lc. lactis* postbiotics. From a public health perspective, these findings are highly significant. For *Lb. plantarum* postbiotics, it is certainly important that they are a strong and natural alternative to antibiotics in bacteria that have developed resistance to many antibiotics, but the fact that *Lc. lactis* postbiotics had only a bacteriostatic effect at the tested concentrations is also quite valuable information. The bacteriostatic effect can be particularly effective in controlling rapidly progressing infections and may help preserve beneficial gut microbiota that are often depleted during aggressive antibiotic treatments. Furthermore, slowing down the growth of pathogens in food poisoning will also give the patient’s immune system time to function. The fact that such natural compounds show strong antimicrobial effects against pathogens that are resistant to many antibiotics is promising. Some postbiotics, such as nisin, are classified GRAS by the FDA. The possibility of postbiotics developing antibiotic resistance or transferring resistance genes is a particularly noteworthy area of research. In this study, purified bacteriocins were not used. However, in analyses of postbiotics, supernatants were pre-treated to eliminate potential effects other than bacteriocins, such as high acidity, low pH, and the antimicrobial effect of hydrogen peroxide. It was determined that the antimicrobial effect originated from one or more proteins of a protein nature. The research results show that postbiotics passed the test in terms of antimicrobial effect, even without using pure bacteriocin. Although the MICs of postbiotics at 60–80% appear relatively high, their application in the poultry industry seems feasible in terms of economic cost, as they would not require purification expenses like bacteriocins. In the food industry, MIC and MBC values are important in many respects in studies on alternative antimicrobial agents. MIC concentrations are important for preventing or delaying microbial spoilage in foods and for extending the existing shelf-life of foods. MBC values are important for ensuring food safety by preventing the growth of pathogens in high-risk foods. In this study, the most influential factors affecting the applicability of the determined MIC and MBC values in the poultry sector are the food matrix and the applied technological processes. Certainly, when it comes to proposing the use of postbiotics as alternative antimicrobial agents in the poultry sector, evaluations are needed through in vivo studies with respect to the relationship between the food matrix and the application technique. Since postbiotics are resistant even at autoclave temperatures, they can be added to the boiling water; they can be used during the spraying stage as they do not cause color or odor changes; or they can be applied directly to meats before packaging. Industry-specific research is needed to determine at what stage, in what dosage, and by what method these postbiotic compounds should be used in the poultry sector.

## 5. Conclusions

The present study highlights the high prevalence of ESBL-positive *E. coli* in chicken meat and underscores the pressing public health challenge posed by antibiotic-resistant foodborne pathogens. Our findings demonstrate that postbiotics derived from *Lb. plantarum* and *Lc. lactis* strains isolated from kefir exhibit significant antimicrobial activity against both reference and ESBL-positive *E. coli* strains. Notably, *Lb. plantarum* postbiotics exerted both bacteriostatic and bactericidal effects, whereas *Lc. lactis* postbiotics showed primarily bacteriostatic activity, emphasizing their potential as natural alternatives to conventional antibiotics. The stability of postbiotic antimicrobial activity under various environmental conditions, including high temperatures, and their effectiveness against multidrug-resistant bacteria, further support their applicability in food safety and public health contexts. While the proportion of effective isolates was limited, the results indicate that postbiotics could be strategically employed in poultry processing to reduce pathogen load, thereby mitigating the risk of foodborne infections. Future studies should focus on optimizing application methods, dosages, and industrial-scale implementation, as well as investigating the mechanisms underlying the differential antimicrobial effects of bacteriocins. Overall, this research provides compelling evidence for the integration of postbiotic-based interventions in the poultry industry, offering a promising and sustainable approach to combat antibiotic-resistant pathogens and enhance food safety.

## Figures and Tables

**Table 1:** Primers used for the identification of lactic acid bacteria.

Bacteria	Primer Sequences	Band Size	References
*Lb. plantarum*	plan F (5′-CCG TTT ATG CGG AAC ACC TA-3′) pREV (5′-TCG GGA TTA CCA AAC ATC AC-3′)	318 bp	[27]
*Lc. lactis*	LacreR (19-GGGATCATCTTTGAGTGAT) LacF (19-GTACTTGTACCGACTGGAT)	161 bp	[28]

**Table 2:** Antimicrobial susceptibility of *E. coli* isolates.

		*E. coli* Isolates (%)	
Antibiotic Group	Antibiotic Agents	Resistant	Susceptible
Penicillin	Ampicillin (10 µg)	100% (*n* = 125)	0% (*n* = 0)
Beta-Lactamase Inhibitor/penicillin	Ticarcillin/Clavulanate (75–10 µg)	30.4% (*n* = 38)	69.6% (*n* = 87)
Cephalosporins	Cefoxitin (30 µg)	100% (*n* = 125)	0% (*n* = 0)
	Ceftriaxone (30 µg)	62.4% (*n* = 78)	37.6% (*n* = 47)
	Cefotaxime (5 µg)	48% (*n* = 60)	52% (*n* = 65)
	Cephalothin (30 µg)	100% (*n* = 125)	0% (*n* = 0)
	Ceftazidime (10 µg)	39.2% (*n* = 49)	60.8% (*n* = 76)
	Cefpodoxime (10 µg)	28% (*n* = 35)	72% (n = 90)
Fluoroquinolones	Ciprofloxacin (5 µg)	40% (*n* = 50)	60% (*n* = 75)
Antimicrobic	Trimethoprim/sulfamethoxazole (125–23.75 µg)	41.6% (*n* = 52)	58.4% (*n* = 73)
	Chloramphenicol (30 µg)	36.8% (*n* = 46)	63.2% (*n* = 79)
Carbapenem	Meropenem (10 µg)	0% (*n* = 0)	100% (*n* = 125)
Monobactam	Aztreonam (30 µg)	100% (*n* = 125)	0% (*n* = 0)
Aminoglycoside	Gentamicin (10 µg)	0.8% (*n* = 1)	99.2% (*n* = 124)
	Amikacin (30 µg)	0.8% (*n* = 1)	99.2% (*n* = 124)
	Netilmicin (10 µg)	10.4% (*n* = 13)	89.6% (*n* = 112)

**Table 3:** Concentration- and time-dependent antimicrobial effects of postbiotics produced by *Lb. plantarum*.

*Lb. plantarum* Postbiotics (Concentration %)
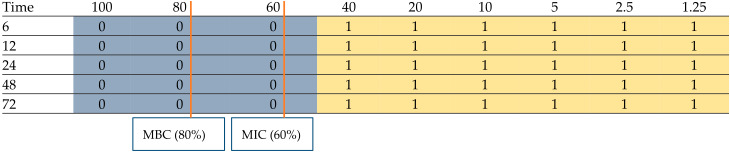

0 = growth not detected, 1= growth detected.

**Table 4:** Concentration- and time-dependent antimicrobial effects of postbiotics produced by *Lc. lactis*.

*Lc. lactis* Postbiotics (Concentration %)
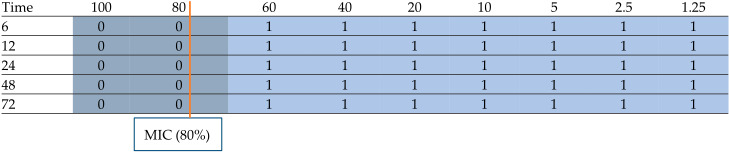

0 = Growth not detected; 1= growth detected.

**Table 5:** Descriptive statistics of the continuous variables used in the study.

Variable	N	Median ± SE	Q1–Q3	Minimum–Maximum
Concentration (%)	6498	20.00 ± 0.43	2.50–60.00	1.25–100
Incubation time (hour)	6498	18.00 ± 0.31	6.00–48.00	0–72

SE: Standard error; Q1–Q3: interquartile range.

## Data Availability

The original contributions presented in this study are included in the article. Further inquiries can be directed to the corresponding author.
